# The relationship between caregiver contribution to self-care and patient quality of life in heart failure: A longitudinal mediation analysis

**DOI:** 10.1371/journal.pone.0300101

**Published:** 2024-03-12

**Authors:** Gabriele Caggianelli, Fabio Alivernini, Andrea Chirico, Paolo Iovino, Fabio Lucidi, Izabella Uchmanowicz, Laura Rasero, Rosaria Alvaro, Ercole Vellone

**Affiliations:** 1 San Giovanni Addolorata Hospital, Rome, Italy; 2 Department of Psychology of Development and Socialization processes, "Sapienza" University of Rome, Rome, Italy; 3 Health Sciences Department, University of Florence, Florence, Italy; 4 Department of Biomedicine and Prevention, University of Rome Tor Vergata, Rome, Italy; 5 Department of Nursing and Obstetrics, Faculty of Health Sciences, Wroclaw Medical University, Wroclaw, Poland; Sant’Anna School of Advanced Studies: Scuola Superiore Sant’Anna, ITALY

## Abstract

**Background:**

Patients with heart failure may experience poor quality of life due to a variety of physical and psychological symptoms. Quality of life can improve if patients adhere to consistent self-care behaviors. Patient outcomes (i.e., quality of life) are thought to improve as a result of caregiver contribution to self-care. However, uncertainty exists on whether these outcomes improve as a direct result of caregiver contribution to self-care or whether this improvement occurs indirectly through the improvement of patient heart failure self-care behaviors.

**Aims:**

To investigate the influence of caregiver contribution to self-care on quality of life of heart failure people and explore whether patient self-care behaviors mediate such a relationship.

**Methods:**

This is a secondary analysis of the MOTIVATE-HF randomized controlled trial (Clinicaltrials.gov registration number: NCT02894502). Data were collected at baseline and 3 months. An autoregressive longitudinal path analysis model was conducted to test our hypotheses.

**Results:**

We enrolled a sample of 510 caregivers [mean age = 54 (±15.44), 24% males)] and 510 patients [mean age = 72.4 (±12.28), 58% males)]. Patient self-care had a significant and direct effect on quality of life at three months (β = 0.20, p < .01). Caregiver contribution to self-care showed a significant direct effect on patient self-care (β = 0.32, p < .01), and an indirect effect on patient quality of life through the mediation of patient self-care (β = 0.07, p < .001).

**Conclusion:**

Patient quality of life is influenced by self-care both directly and indirectly, through the mediation of caregiver contribution to self-care. These findings improve our understanding on how caregiver contribution to self-care improves patient outcomes.

## 1 | Introduction

Heart failure (HF) is a complex disorder characterized by anatomical and functional defects that prevent the heart from efficiently contracting and releasing [1]. HF is regarded as an increasing global pandemic, affecting more than 60 million people globally [2], with approximately 6 million in the United States and 15 million in Europe [3]. Notwithstanding, the prevalence of HF is projected to rise as a result of an ageing population, improved risk factors management and rising survival rates [4, 5].

The effects of HF on health continue to be severe despite the availability of novel therapeutic techniques [6]. HF is linked to higher rates of hospitalization and higher rates of morbidity and mortality, which puts a heavy social and financial strain on healthcare systems [3].

HF physical and psychological symptoms such as dyspnea, fatigue, and depression are the most common reasons for hospitalization and utilization of emergency services [7, 8]. Furthermore, they can have a significant impact on quality of life (QOL) [9], which is strongly associated with adverse HF-related clinical outcomes [10]. Self-care can be a strategy to improve symptoms and as a result, QOL.

HF self-care is defined by Riegel et al., [11] as the efforts made by patients to maintain a stable disease state as well as monitor and manage symptoms. Self-care includes a number of behaviors ranging from adherence to medications, dietary regimens, and physical exercise, to interaction with health care providers. Such behaviors are important because they often lead to symptoms’ reduction, and prevention of HF exacerbations [12]. Consequently, self-care in HF has the potential to reduce rehospitalizations and mortality rates while enhancing overall QoL [13].

Unfortunately, for most HF patients, self-care is an arduous task to follow; Specific factors such as older age, cognitive impairment, comorbidities, psychological distress, and living conditions (e.g., living alone) are found to contribute to self-care inadequacy in this population [6] In this context, the contribution of the caregivers is crucial in promoting better self-care behaviors, and ultimately accomplishing better health outcomes [14].

Caregiver contribution to HF self-care is defined as the process by which the informal caregiver (e.g., a family member) suggests or performs for the patient those activities that promote HF stability (CC to self-care maintenance), facilitate HF symptom monitoring and perception (CC to symptom monitoring and perception), and manage HF symptoms and signs of decompensation (CC to self-care management) [15]. From both a theoretical and empirical point of view, greater involvement of caregivers in self-care promotion can improve outcomes of HF individuals [16]. Considering the intimate connection between HF patients and their caregivers, an increased CC to self-care might be helpful in managing the symptoms of the disease and prompting adherence to the recommended treatment plan [17]. As a result, patient overall QOL, number of hospital admissions, and survival could all improve [18].

Although there is proof of better patient outcomes driven by the support of their caregivers, up to date, there is no evidence of the mechanism by which this relationship unfolds. More specifically, we do not know if HF patient QOL improves as a direct effect of the caregiving influence or rather, indirectly, through the influence of patient self-care. Knowing this mechanism would be beneficial from both a clinical and theoretical standpoint, in terms of educational intervention priorities. Therefore, the aim of this study was to investigate the influence of CC to self-care on QOL in people with HF (distal outcome), and to explore whether patient self-care (proximal outcome) mediates such a relationship.

## 2 | Methods

### 2.1 | Study design

We conducted a secondary analysis on data from participants of the MOTIVATE-HF randomized controlled trial, whose purpose was to improve HF patients’ self-care through motivational interviewing [19]. The results are reported elsewhere, however briefly, MOTIVATE-HF demonstrated that motivational interviewing could improve self-care and QoL, and reduce physical symptoms and all-cause mortality [20–22]. For the present study, we used data from the MOTIVATE-HF trial collected at baseline and 3-month follow-up.

### 2.2 | Sample, setting, and procedure

A sample of 510 patient and caregiver dyads was consecutively recruited from three different cardiology settings in central Italy between June 2014 and October 2018. Participants were randomly assigned to one of three arms: Arm 1, in which MI was used just for patients, Arm 2, in which MI was used for patients and their informal caregivers, and Arm 3, in which patients received standard of care.

Patients were eligible if they had HF, were in a New York Heart Association (NYHA) functional class between II and IV, had poor self-care, defined as a score of 0, 1 or 2 on at least two items on the Self-Care Maintenance or Self-Management scales of the Self-Care of HF Index v.6.2 (SCHFI v.6.2) [23], and were willing to participate in the study. Patients were excluded if they had had a myocardial infarction in the previous 3 months, lived in residential facilities, and had severe cognitive impairment, defined as a score of 0 to 4 on the Six-Item Screener [24].

Caregivers were enrolled if patients recognized them as those who took most of the responsibility of the informal care. If one member of the dyad refused to participate in the study at the time of enrolment, both participants were excluded.

The MOTIVATE-HF study [25] was approved by the Ethics Committee of the University of Rome Tor Vergata (Italy) under protocol 0015133/2013. Before joining the study, patients and caregivers were informed about the procedures and that their personal information was kept anonymous. They were then invited to sign the informed consent form. Data were collected from the 3^rd^ of June 2014 to the 20^th^ of October 2018. This research was conducted in accordance with the principles of the Declaration of Helsinki [26].

### 2.3 | Measures

*CC to patient self-care* was measured with the Caregiver Contribution to Self-Care of HF index (CC-SCHFI) [27] The CC-SCHFI is a valid and reliable instrument consisting of 22 items distributed across 3 distinct scales to measure CC to self-care maintenance (10 items), CC to self-care management (6 items), and caregiver confidence (6 items). For simplicity of our analysis, we only used the CC to self-care maintenance scale. Each item on this scale has a 4-point rating scale format, from 1 = never to 4 = always or daily. The total score is standardized 0–100, with higher scores indicating better CC to self-care maintenance. A score ≥70 is considered adequate CC to self-care [27].

*Patient self-care* was evaluated with the Self-Care of Heart Failure Index (SCHFI v.6.2) [23]. This instrument has 22 items, as the CC-SCHFI is distributed into three distinct scales to measure self-care maintenance, management, and confidence. A 4-point rating scale format is used for each SCHFI scale, from 1 = never or rarely to 4 = always or daily. For this study we only used the self-care maintenance scale, which has a standardized score from 0 to 100, with higher score meaning better self-care maintenance behaviors. A score ≥70 is considered adequate self-care [23].

*QOL* was measured with the 12-item Short-Form Health Survey (SF-12) [28]. This scale consists of 12 items divided into two scales: physical component score (PCS-12) and mental component score (MCS-12). The PCS items concern physical QOL, influenced by physical health. The MCS items concern mental QOL, influenced by mental health. All scores are standardized 0–100, with higher scores indicating better mental and physical QOL [28]. As previously done by other authors [29], we used a single score of QOL reflecting both its physical and mental dimensions; this was adopted to simplify the analysis, and in order to have a general QOL estimate.

### 2.4 | Statistical analysis

To describe the sociodemographic characteristics of the sample, we used descriptive statistics. We calculated means and standard deviations for continuous variables, and percentages and frequencies for categorical variables. Normal distribution assumption of the variables was examined with skewness and kurtosis, with acceptable values ranging between -2 and +2 [30].

A longitudinal autoregressive path analysis model was tested with the following variables: (i) CC to self-care at three months; (ii) patient self-care at three months (mediator); (iii) QOL at three months (dependent variable); (iv) # two dummy variables coding the interventions. The first with a value of “1” indicating people who participated in intervention on patient in MOTIVATE-HF [19] and a value of “0” for people who did not participate. The second dummy with a value of “1” indicating people who participated in intervention on dyads in MOTIVATE-HF and a value of “0” for people who did not participate.

The advantage of adopting a longitudinal approach, compared to cross-sectional models, is that the statistical effects are estimated taking into account the baseline level of the variables, above and beyond the information contained in that starting level. This approach makes more data available to establish the temporal antecedence of the posited relationships in the model [31].

The mean score of each scale was computed and used in the analysis. As regards testing mediators, the confidence intervals (CIs) for the hypothesized indirect effects were calculated using the Delta method [32]. The goodness of fit of the model was judged with conventional criteria, employing both the chi-square test statistic and the following fit indices: the Comparative Fit Index (CFI) and the Tucker–Lewis index (TLI), both of which should be higher than .90 in a good-fit model [33], the Root Mean Square Error of Approximation (RMSEA) and the Standardized Root Mean Square Residual (SRMR), which are expected to be lower than .08 in an acceptable fit [34]. Statistical analyses were performed using Jamovi v.2.2 [35].

## 3 | Results

### 3.1 | Characteristics of participants

Table 1 summarizes the characteristics of patients and their caregivers. Caregivers had a mean age of 54 years (±15.44), were predominantly female (74.5%), married (70.8%), employed (73.5%), and lived with their patients (60%). CC to self-care maintenance scores averaged 51.48 years (±19.69) (Table 2) indicating insufficient contribution to self-care.

**Table 1:** Baseline sociodemographic characteristics of the sample.

*Characteristics*	Caregivers (n = 510)	Patients (n = 510)
Age, mean (SD)	53.97 (15.44)	72.37 (12.28)
Sex (*male*), n (%)	123 (24.1)	296 (58)
Marital Status, n (%)		
*Married*	361 (70.8)	316 (62)
*Widower*	12 (2.4)	150 (29.4)
*Divorced*	36 (7.1)	20 (3.9)
*Single*	93 (18.2)	24 (4.7)
Education level (*high school or higher*), n (%)	275 (53.9)	132 (25,9)
Employment (*retired*), n (%)	135 (26.5)	387 (75.6)
Live with patient, n (%)	306 (60)	-
Relationship with the patient, n (%)		
*Spouse*	189 (37.1)	-
*Child*	196 (38.4)	-
*Other*	118 (23.1)	-
NYHA class, n (%)		
*II*	-	313 (61.4)
*III*	-	160 (31.4)
*IV*	-	33 (6.5)

SD, standard deviation; HF, heart failure; NYHA, New York Heart Association.

**Table 2:** Scores of the variables at baseline and three months follow-up.

	**Baseline (T0)**	
*Characteristics*	Caregivers	Patients
SF-12 total score, mean (SD)		
* PCS-12*	-	44.74 (10.17)
* MCS-12*	-	35.46 (9.57)
Self-Care of HF Index, mean (SD)		
* Self-Care maintenance*	-	45.55 (15.39)
CC to Self-Care of HF Index, mean (SD)		
* CC to Self-Care maintenance*	51.48 (19.69)	-
	**Three months (T1)**	
*Characteristics*	Caregivers	Patients
SF-12 total score, mean (SD)		
* PCS-12 (n = 146 missing)*	-	46.09 (9.61)
* MCS-12 (n = 146 missing)*	-	37.27 (9.23)
Self-Care of HF Index, mean (SD)		
* Self-Care maintenance (n = 147 missing)*	-	52.12 (20.42)
CC to Self-Care of HF Index, mean (SD)		
* CC to Self-Care maintenance (n = 191 missing)*	54.52 (20,63)	-

SD, standard deviation; SF, Short Form; PCS, physical component score; MCS, mental component score; SCHFI, Self-Care of HF Index; CC, caregiver contribution.

Patients had a mean age of 72.4 (±12.28) years, were mostly male (58%), retired (76.2%) and in NYHA class II (61.4%) (Table 1). Mean baseline self-care maintenance scores averaged 45.55 (±15.39), indicating insufficient self-care. QOL was poor in both their mental and physical components, with mean baseline scores of 35.46 (±10.17) and 44.74 (±10.17), respectively (Table 2).

### 3.2 | Influence of CC to self-care on patient QOL

**Fig 1:**
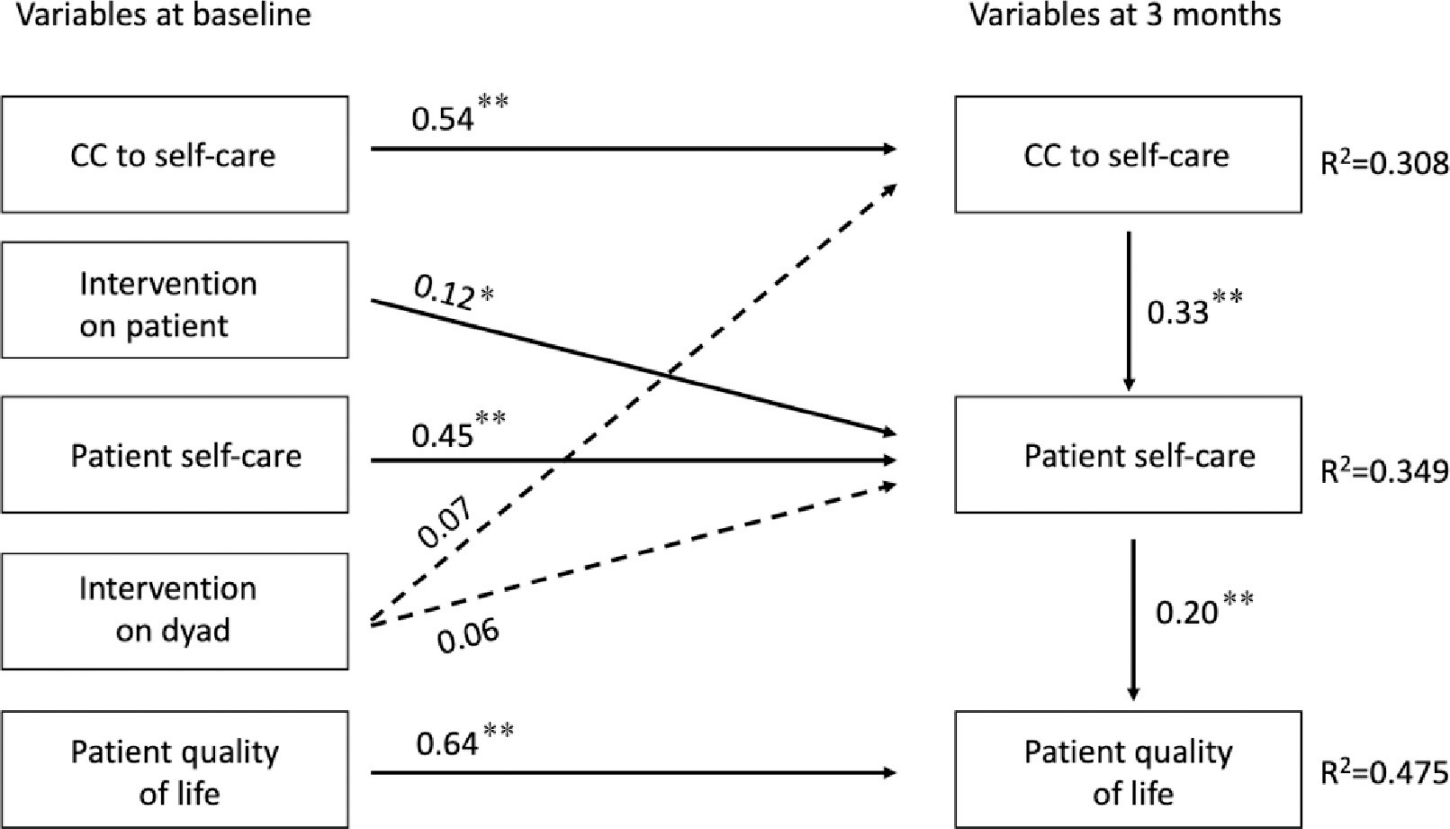
Path diagram depicting the influence of caregiver contribution to self-care on patient quality of life. **Note.** Dotted arrows indicate non-significant paths. All coefficients come from a complete standardized solution. * = p < .05; ** = p < .01; *** *** = p < .001.

The skewness and kurtosis of the variables ranged between 0.08 and 0.5, indicating a normal distribution of the scores. The path analysis (Fig 1) exhibited the following goodness of fit indexes: χ^2^ [14] 31, p = .006; CFI 0.97; TLI 0,94; RMSEA 0.07; SRMR 0.04. We found that CC to self-care (at baseline) had a direct effect on patient self-care (at 3 months) (β = 0.32, p < .01) and in turn patient self-care (at 3 months) had a direct effect on patient QOL (at 3 months) (β = 0.20, p < .01). In addition, we found that CC to self-care had a significant indirect effect on patient QOL through the mediation of patient self-care (β = 0.07, p < .001) (Table 3). We found no direct effect of CC to self-care on patient QOL (p>.05). The model accounted for 31% of the variance in CC to self-care, 35% of variance of patient self-care, and 47% in patient QOL (T1).

**Table 3:** Indirect effects estimated in the model.

Description	Estimate (β)	SE	p
CCT0⇒ CCT1 ⇒ PSCT1 ⇒ PQOLt1	0.036	0.009	< .001
CCT0⇒ CCT1 ⇒ PQOLT1	0.032	0.023	0.21
Intervention on dyad ⇒ CCT1 ⇒ PSCT1 ⇒ PQOLT1	0.004	0.004	0.256
Intervention on dyad ⇒ CCT1 ⇒ PQOLT1	0.003	0.004	0.392
Intervention on dyad ⇒ PSCT1 ⇒ PQOLT1	0.014	0.014	0.243
Intervention on PT ⇒ PSCT1 ⇒ PQOLT1	0.025	0.014	0.03
CCT1 ⇒ PSCT1 ⇒ PQOLT1	0.067	0.015	< .001
PSCT0 ⇒ PSCT1 ⇒ PQOLt1	0.091	0.024	< .001

CCT0 = Caregiver Contribution to self-care at baseline; CCT1 = Caregiver Contribution to self-care at 3 months; PSCT0 = Patient Self-Care at baseline; PSCT1 = Patient Self-Care at 3 months; PQOLT1 = Patient Quality of Life at 3 months.

## 4 | Discussion

The aim of this study was to investigate the influence of CC at self-care on QOL in patients with HF and explore whether patient self-care mediated this relationship. We found that CC at self-care influences patient QOL indirectly through the mediation of patient self-care. To our knowledge, this is the first study that examined the mechanism by which caregiver support improves QOL in patients with HF.

Our study demonstrates a significant relationship between CC to self-care and patient self-care. In accordance with theory, CC to self-care can be seen as a “support” that caregivers offer to patients, such as reminding patients to take their medications, or as actions that prompts the patients to act, such as alerting the patient to consult his/her healthcare provider when presenting symptoms [15]. Therefore, CC to self-care and patient self-care are inextricably interrelated in that CC to self-care influences patient self-care, which in this case can be operationalized as a proximal outcome. Prior studies had already found that “generic” social support from caregivers improves patient self-care [36], but in this study we have proven for the first time that patient self-care is directly improved by CC to self-care. Indeed, this could be expected but no study had proved this relationship before. This evidence suggests that people close to the patient, such as the family, relatives, or friends, have a fundamental role in encouraging patients to improve their self-care behaviors and, consequently, protect them from factors that might worsen HF [19]. This result further supports the hypothesized theory that caregiving behaviors have the potential to improve patients’ outcomes. When caregivers prioritize self-care for their loved ones, they can benefit in terms of improved physical, mental and emotional health [37].

We observed that patients having a higher engagement in self-care resulted in a direct benefit on their QOL. Studies on the relationship between patient self-care and QOL are inconsistent; some have found that better patient self-care is associated with better QOL [38, 39], as we found, but other studies did not find a significant relationship between self-care and QOL [40, 41]. This lack of consistency in the literature could be justified by the fact that self-care behaviors in HF have the potential to improve QOL, but sometimes patients perform self-care only when they have symptoms, the disease is in an advanced stage and their QOL is very poor.

In the present study we demonstrated that CC to self-care influences patient QOL through the mediation of patient self-care. Although this effect could be expected this was never objectively proven. This finding is important because it indicates that the mechanism by which CC to self-care improves QOL is by improving patient self-care. In other words, caregivers improve the self-care of their patients, and this improvement ameliorates patient QOL. This finding is important from a clinical and theoretical perspective. From a clinical perspective, our finding supports the importance to develop interventions aimed also at improving CC to self-care, because by improving CC to self-care, indirectly, also patient QOL improves; from a theoretical perspective our finding can add new elements to the situation-specific theory of caregiver contribution to self-care [15]. This theory has generically identified positive and negative outcomes for caregivers and patients, but now we can be more specific by affirming that patient self-care is another outcome of the theory.

### 4.1 | Strengths and limitations

Strengths of this study include the longitudinal and theoretically driven statistical analysis, which confers higher possibility to infer causal relationships. However, the analysis was based on data from a mono-national clinical trial and the patients involved in the study were mainly in NYHA class II and III, which restrict the generalizability of the findings. While our study design aimed for a robust research approach, it is important to note our results do not explicitly control for certain potential confounding factors. Specifically, variations in caregiver characteristics, intensity and nature of caregiver interventions, and the degree of patient receptivity to caregiver support were not systematically accounted for in our analysis. These factors may introduce external influences that could impact the observed relationship between variables. Overall, we expect an average variation of these factors normally distributed as the other variables of our study.

## 5 | Conclusion

Our study demonstrates the important role of informal caregivers in improving patient self-care and patient QOL. This new knowledge might have practical implications for designing future interventions, because healthcare providers should not only consider HF patients but also their caregivers in a caring context. Also, this study advances the situation-specific theory of caregiver contribution to self-care which is now more precise in its outcome part.

## Supporting information

S1 Dataset (CSV)
